# Engineering *Penicillium expansum* antifungal proteins unveils new clues about their mode of action

**DOI:** 10.1007/s00253-026-13782-5

**Published:** 2026-04-20

**Authors:** Moisés Giner-Llorca, Francisca Gallego del Sol, Stefani de Ovalle, Darren D. Thomson, Elaine M. Bignell, Alberto Marina, Jose F. Marcos, Paloma Manzanares

**Affiliations:** 1 Department of Food Biotechnology, Instituto de Agroquímica y Tecnología de Alimentos (IATA), Consejo Superior de Investigaciones Científicas (CSIC), Paterna, Spain; 2 Instituto de Biomedicina de Valencia (IBV), CSIC and CIBER de Enfermedades Raras (CIBERER), Valencia, Spain; 3 Department of Biosciences, Faculty of Health and Life Sciences, Medical Research Council Centre for Medical Mycology at the University of Exeter, Exeter, UK

**Keywords:** Antifungal proteins (AFPs), Rational design, Protein internalisation, Reactive oxygen species (ROS), Membrane permeabilisation, γ-core motif

## Abstract

**Abstract:**

Fungal antifungal proteins (AFPs) are promising biofungicides. PeAfpA and PeAfpB from *Penicillium expansum* show different activity profiles and potency, with PeAfpA being more active. Based on the PeAfpB solved structure, we had previously designed PeAfpB-PeAfpA chimeras that showed different properties. From these, we engineer here two additional variants, chPeAFPV6 and chPeAFPV7, that revealed novel aspects of the AFP structure, antifungal determinants and mechanism. chPeAFPV6, with a single E11K mutation in the loop L1 that is part of the γ-core motif, increased PeAfpB antifungal activity to that of PeAfpA against filamentous fungi but not yeasts, and promoted internalisation into *Penicillium digitatum* hyphae*.* However, changes in loop L3 of PeAfpB as in chPeAFPV7 abolished this increase, resulting in an inactive protein that still internalised. Overall, internalisation is neither sufficient nor essential for killing *P. digitatum*. Antifungal activity did not correlate with reactive oxygen species production, suggesting that oxidative burst is a fungal stress defence rather than a killing mechanism. Although cell permeabilisation was associated with antifungal activity, it does not seem to be a primary mode of action. Structural analysis showed interactions between the γ-core motif and loop L3, and suggests the importance of the conformation of the E7 residue of PeAfpB. Additionally, PeAfpA was identified as a protein able to penetrate *Candida auris* by a cell wall-dependent mechanism, and kill yeast cells. This study highlights the potential of the PeAfpB scaffold for engineering new-to-nature AFPs and provides novel insights into their modes of action, paving the way for future applications.

**Key points:**

- *A single amino acid change in the γ-core of PeAfpB enhances antifungal potency*
- *Loop L3 of PeAfpB may block activity through interaction with the γ-core*
- *Antifungal activity does not correlate with ROS production*

**Supplementary Information:**

The online version contains supplementary material available at 10.1007/s00253-026-13782-5.

## Introduction

Filamentous fungi pose a serious threat to food security worldwide due to their ability to infect a vast range of crops, causing significant economic losses (Fisher et al. 2020). Moreover, fungal pathogens are a major concern for human health, with *Aspergillus* and *Candida* species being the most common cause of systemic fungal infections (Kubota et al. 2023). Options available to combat fungal infections are limited to a few classes of molecules such as azoles, echinocandins, polyenes and pyrimidine analogues (Chen et al. 2023). However, there is an increasing emergence of fungicide-resistant isolates and cross-resistance is appearing between antifungals used in agriculture or clinic (Fisher et al. 2022, 2018; Perfect 2016), making it urgent to develop novel biofungicides with modes of action different from the existing ones.

Antifungal proteins (AFPs) produced by filamentous ascomycetes are small, cationic and cysteine-rich proteins (CRPs) with high potency against a broad spectrum of plant and human fungal pathogens (Hegedus and Marx 2013; Lacadena et al. 1995; Martínez-Culebras et al. 2021; Meyer 2008). AFPs contain six to eight cysteine residues that form three or four intramolecular disulphide bonds stabilising an antiparallel β-sheet structure with surface-exposed loops (Batta et al. 2009; Campos-Olivas et al. 1995; Hajdu et al. 2019; Huber et al. 2018). Due to this compact structure and the stabilising pattern of disulphide bonds, these proteins show high stability against pH changes, high temperature and proteolytic treatment (Batta et al. 2009). These highly desirable physicochemical properties, their ability to protect plants, crops and fruits from fungal attack (Bugeda et al. 2025; Gandia et al. 2021, 2020; Garrigues et al. 2018; Toth et al. 2020; Tóth et al. 2022) and their lack of toxicity against plants or animals (Matejuk et al. 2010; Szappanos et al. 2006, 2005; Vila et al. 2001) make them appropriate candidates for developing new antifungals.

Some fungal genomes encode more than one AFP. For example, the pome fruit pathogen *Penicillium expansum* encodes three AFPs that belong to different phylogenetic classes: PeAfpA, PeAfpB and PeAfpC (Garrigues et al. 2018, 2016). Among them, PeAfpA is one of the most active AFPs against several plant and human pathogens, PeAfpB showed a moderate activity against most of the mycotoxigenic fungi tested, while PeAfpC was inactive against most of the fungi evaluated (Garrigues et al. 2018; Giner-Llorca et al. 2023a; Martínez-Culebras et al. 2021). Moreover, PeAfpA shows protective effect to control fruit decay (Bugeda et al. 2025; Gandia et al. 2021; Garrigues et al. 2018) and is successfully produced in safe fungal biofactories (Gandía et al. 2022).

Regarding the PeAfpA mode of action, we have shown that chitin synthesis, protein O-mannosylation and interaction with phospholipids are relevant factors (Gandia et al. 2019; Giner-Llorca et al. 2023a). In addition, the cell wall integrity (CWI) pathway is a key player in the PeAfpA killing mechanism against *Saccharomyces cerevisiae* (Giner-Llorca et al. 2023b). Recently, a study focused on the dynamics of PeAfpA interaction and internalisation in *Penicillium digitatum* morphotypes (Giner-Llorca et al. 2024). The protein binds to the conidial wall in a distinctive pattern that changes with germination but is not internalised. PeAfpA preferentially interacts with hyphal tips of growing mycelium and is rapidly internalised via an energy-dependent process, causing vacuolisation and cell death (Giner-Llorca et al. 2024). Finally, it was also demonstrated that PeAfpA killing action does not rely on the induction of reactive oxygen species (ROS) (Giner-Llorca et al. 2024).

We previously reported the 3D structure of PeAfpB solved by X-ray crystallography and the rational design of five PeAfpB-PeAfpA chimeras (chPeAFPV1-V5), in which high variable residues from loops connecting the core β-strands and the C-terminal end of the PeAfpB sequence were substituted by those corresponding to PeAfpA (Giner-Llorca et al. 2023a). Chimeras showed a wide range of antifungal activity, from that more similar to PeAfpA like chPeAFPV1 to almost total loss of activity like chPeAFPV3. Moreover, the analysis of chimeric structures showed an almost identical conformation to the PeAfpB structure, suggesting that the differences in activity are due to the contributions of specific residues.

With the aim of identifying the contributions of different sequence motifs to the mode of action, in the present study we report the rational design of two new PeAfpB-PeAfpA proteins (chPeAFPV6-V7) based on the most relevant chimeras previously described, the highly active chPeAFPV1 and the inactive chPeAFPV3. We have characterised the newly designed proteins and solved their 3D structure by X-ray crystallography. Moreover, the role of cell permeabilisation and ROS production on the mode of action of parental and chimeric AFPs is investigated. In addition, the interaction and internalisation pattern of these proteins in two target fungi, *P. digitatum* and *Candida auris*, is presented.

## Materials and methods

### Strains, media and culture conditions

*Penicillium chrysogenum* ∆*paf* strain (Hegedus et al. 2011) was used for genetic transformation and recombinant protein production. For antimicrobial activity assays, the following fungal strains were used: *P. expansum* CECT 20906 (CMP-1), *P. digitatum* CECT 20796 (PHI-26), *P. chrysogenum* Q176, *Penicillium roqueforti* CECT 2905, *Botrytis cinerea* CECT 20516, *Byssochlamys spectabilis* CECT 2983, *Aspergillus westerdijkiae* CECT 2948, *Fusarium oxysporum* 4287, *Magnaporthe oryzae* PR9, *Saccharomyces cerevisiae* BY4741, *Aspergillus fumigatus* ATCC 46645, *Cryptococcus neoformans* H99, *Candida albicans* SC5314, *Candida glabrata* 4012, *C. glabrata* 1384, *C. auris* B121040, *C. auris* B12406, *C. auris* B12663 and *C. auris* B17201. Filamentous fungi were cultured in Potato Dextrose Agar (PDA) plates at 25 °C for 5–10 days, except for *A. fumigatus,* which was grown at 37 °C for 3–4 days. *S. cerevisiae* was grown in Yeast Extract Peptone Dextrose Agar (YPDA) plates at 30 °C for 2 days. *C. neoformans* and *Candida* spp. were cultured in RPMI-1640 (Gibco) at 37 °C and 200 rpm for 24 h. The work on human fungal pathogens was carried out based on the biosafety guidelines described in the Control of Substances Hazardous to Health (COSHH) regulations.

For fungal transformation, vectors were propagated in *Escherichia coli* JM109 or *Agrobacterium tumefaciens* AGL-1 as previously described (Giner-Llorca et al. 2023a).

### Production, purification and identification of recombinant proteins

Genetic elements from the FungalBraid (FB) modular cloning platform (Hernanz-Koers et al. 2018) previously available or generated in this study are described in Table S1 and Fig. S1. Molecular assemblies used to produce the recombinant proteins were generated and confirmed as previously described (Giner-Llorca et al. 2024; Hernanz-Koers et al. 2018) (Table S1). Binary assemblies generated for fungal transformation were introduced into *A. tumefaciens* AGL-1 strain by electroporation. *P. chrysogenum* ∆*paf* strain was genetically transformed through *A. tumefaciens*-mediated transformation (ATMT) (Harries et al. 2015; Vazquez-Vilar et al. 2020). *P. chrysogenum* transformants were selected and confirmed by PCR (Table S2; Fig. S1) (Khang et al. 2006).

Production, purification and identification of PeAfpA, PeAfpB and variants were performed as described (Garrigues et al. 2018; Giner-Llorca et al. 2024).

### Protein crystallisation and data collection

Crystals from the newly designed proteins chPeAFPV6 and chPeAFPV7 were grown in sitting drops at 21 °C using the vapour-diffusion method. Initial crystallisation trials were set up in the Crystallogenesis service of the IBV-CSIC using commercial screens JBS I, JBS II (JENA Biosciences) and JCSG (Molecular Dimensions) in 96-well plates (Swissci MRC2) using equal volumes of protein at 40 mg/mL in buffer A (25 mM Tris buffer pH 8, 200 mM NaCl) and mother liquor. chPeAFPV6 and chPeAFPV7 crystals grew in 7 days using 1.6 M ammonium sulphate and 1 M of lithium sulphate as mother liquor. Diffraction data were collected at 100 K from single crystals at the XALOC beamline of ALBA Synchrotron. Data sets were processed using XDS (Kabsch 2010) and reduced with Scala (Evans 2006) (CCP4). The data-collection statistics are shown in Table S3.

### Phase determination, model building and refinement

Crystal structures of chPeAFPV6 (PDB ID: 9FQF) and chPeAFPV7 (9FQG) were solved by molecular replacement with MOLREP (Vagin and Teplyakov 2010) using PeAfpB coordinates (7ZTF) as the search model. The final models were refined by alternating several rounds of manual building with COOT (Emsley and Cowtan 2004) and automatic refinement with REFMAC (Vagin et al. 2004). Programs used are included in the CCP4 Cloud package (Agirre et al. 2023). Refinement statistics are summarised in Table S3. Figures for structural representations were generated with PyMOL software (https://pymol.org/2, Schrödinger) and UCSF Chimera X software (Goddard et al. 2018).

### Antimicrobial activity assays

Fungal growth inhibition assays were conducted in 96-well microtiter plates (Nunc). Antifungal activity against fungal conidia was carried out as described (Giner-Llorca et al. 2023a). Dose–response curves were generated from measurements after 48 h in yeasts, and 72 h in filamentous fungi. Minimum inhibitory concentration (MIC) is defined as the protein concentration that completely inhibited growth in all the experiments performed. To evaluate antifungal activity against germlings, conidia were allowed to germinate by incubation for 16 h at 25 °C and assays performed as previously described (Giner-Llorca et al. 2024).

### SYTOX Green uptake assay

SYTOX Green (SG) uptake by *P. digitatum* mycelium after protein treatment was determined by fluorometric measurement using a microplate assay. One hundred and fifty µL of conidia (10^6^ conidia/mL) in 10% PDB containing 0.02% (w/v) chloramphenicol were statically incubated for 16 h at 25 °C. Then, 30 µL of 6.66 × concentrated AFP (from serial twofold dilutions) and 20 µL of 10 × concentrated SG (Invitrogen) (0.2 µM, final concentration) were added to each well. Protein final concentrations range from 0.125 to 32 µg/mL (0.02 to 4.8 µM). Samples were prepared in triplicates and the experiment was repeated three times. After the addition of proteins and SG, plates were statically incubated for 8 h at 25 °C and fluorescence intensity was acquired using a microplate reader (CLARIOstar, BMG Labtech). Excitation and emission wavelengths were set to 480 ± 10 nm and 530 ± 10 nm, respectively. Fluorescence values were calculated by subtracting control data (no protein added) and then normalised by dividing by the growth (OD600). Finally, mean and standard deviation (SD) from triplicate values were calculated.

### Determination of ROS

ROS levels on AFP-treated germlings were determined by fluorometric measurement as described (Giner-Llorca et al. 2024). Protein final concentrations ranged from 0.5 to 32 µg/mL. Samples of 50 mM H_2_O_2_-treated germlings were included as controls of inhibition caused by oxidative stress. After the addition of proteins and the ROS probe CM-H_2_DCFDA, plates were statically incubated for 8 h at 25 °C and fluorescence intensity was acquired at 1, 2, 4 and 8 h (excitation and emission wavelengths set to 490 ± 10 nm and 530 ± 20 nm, respectively). Fluorescence values were calculated by first subtracting control data (no protein added) and then normalised by dividing by the growth (OD600). Finally, mean and standard deviation (SD) from triplicate values were calculated.

### Confocal microscopy

AFPs were fluorescently labelled with BODIPY™ FL EDA (Invitrogen) as described (Giner-Llorca et al. 2024). Labelled proteins were quantified by the bicinchoninic acid assay (Sigma Aldrich) and their in vitro antifungal activity was confirmed.

The interaction of BODIPY™ labelled AFPs with *P. digitatum* CECT 20796 growing hyphae and conidia was analysed by confocal microscopy following previously described protocols (Giner-Llorca et al. 2024). In experiments with *C. auris* B121040, a final concentration of 5 × 10^6^ cfu/mL in PDB 5% was used and AFPs added at a final concentration of 32 µg/mL. Cell wall (CW) was stained with 50 µg/mL CFW (Fluorescent Brightener 28; Sigma-Aldrich) for 15 min. Propidium iodide (PI) at 20 µg/mL (Sigma-Aldrich) was used to analyse cell viability. In these experiments, labelled-AFPs were added first, followed by the addition of CFW and PI. Confocal images of conidia were acquired at the Central Service for Experimental Research of the University of Valencia (SCSIE-UV), using a Zeiss LSM 980 confocal laser scanning microscope and with a 63 × oil immersion objective (63/1.4NA PlanApo DIC, Zeiss). Confocal images of growing hyphae and yeasts were acquired at the MRC of the University of Exeter, using an Andor DragonFly spinning disk confocal microscope (Oxford Instruments) with a 60 × oil immersion objective (60/1.49NA PlanApo TIRF, Nikon) as described (Giner-Llorca et al. 2024). Labelled AFPs were excited at 488 nm and detected at 521 ± 38 nm. CFW was excited at 405 nm and detected at 445 ± 46 nm. PI was excited at 561 nm and detected at 594 ± 43 nm.

## Results

### Rational design, production and activity of chimeric proteins

**Fig. 1:**
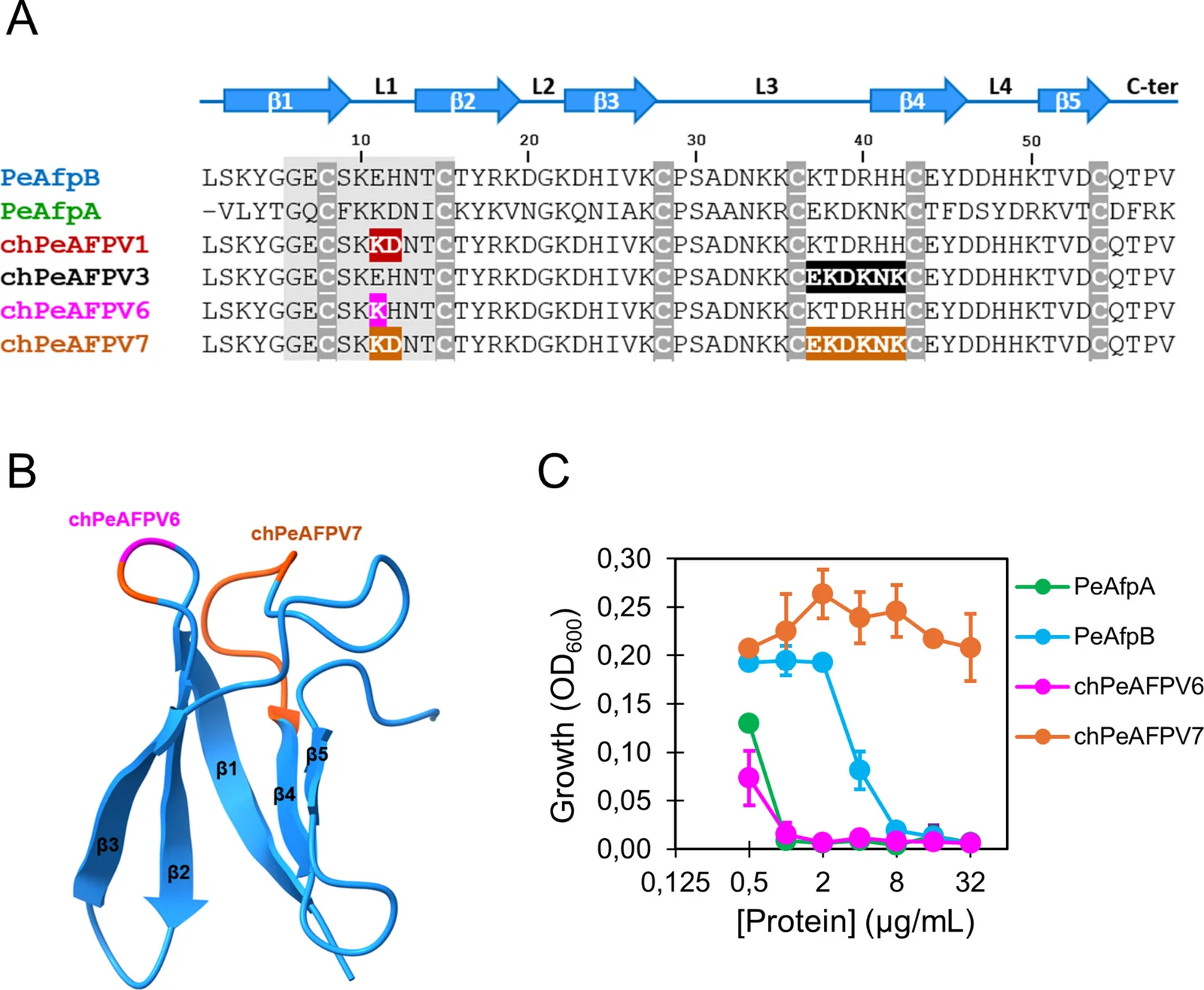
Design of chPeAFPs. **A** Sequence alignment of PeAfpB, PeAfpA, chPeAFPV1, chPeAFPV3 and the newly designed chPeAFPV6 and chPeAFPV7. Amino acids exchanged between PeAfpB and PeAfpA to generate the chimeras are highlighted in red for chPeAFPV1, black for chPeAFPV3, magenta for chPeAFPV6 and orange for chPeAFPV7. The conserved ɣ-core motif is represented as a grey box. At the top of the alignment is shown the PeAfpB secondary structure with arrows and lines for β-sheets and coiled regions, respectively. Cysteine residues are underlined and highlighted in grey. The structures of PeAfpB (PDB ID: 7ZTF), chPeAFPV1 (7ZUT) and chPeAFPV3 (7ZVH) have been solved previously (Giner-Llorca et al. 2023a). **B** Cartoon representation of PeAfpB structure using the same colour code as in (A) to visualise the location of the substitutions. **C** Dose–response curves showing the inhibitory activity of AFPs against *P. digitatum* conidia

In our previous work, we validated PeAfpB as a scaffold for improved design and production of new-to-nature AFPs and designed five PeAfpB-PeAfpA chimeric proteins (chPeAFPV1-V5) (Giner-Llorca et al. 2023a). In chPeAFPV1, the PeAfpB E11 and H12 residues located in loop L1 within the conserved γ-core motif were substituted for the PeAfpA K10 and D11 residues, while chPeAFPV3 resulted from a six residues substitution between C36 and C43 corresponding to the second part of the long loop L3 and the initial part of β4 strand (Fig. 1A). Notably, the changes in chPeAFPV1 enhanced the antifungal potency of PeAfpB, making it similar to that of PeAfpA for several phytopathogenic fungi. By contrast, chPeAFPV3 was inactive towards most of the fungi tested, pointing out the critical importance of residues 37–42 in the antifungal activity of PeAfpB and the existence of at least two critical motifs separated in the protein sequence but proximal in the 3D structure (Fig. 1B).

With the aim of further investigating the relevance of residues KD and EKDKNK in the antifungal activity and mode of action as well as any potential interaction between these motifs, we designed two new PeAfpB-PeAfpA proteins, chPeAFPV6 and chPeAFPV7 (Fig. 1A-B). chPeAFPV6 resulted from the substitution of the single PeAfpB residue E11 for the PeAfpA residue K10, while chPeAFPV7 derived from the combination of chPeAFPV1 and chPeAFPV3. chPeAFPV6 was designed to evaluate whether a single amino acid change within the γ-core could improve antifungal activity, while chPeAFPV7 modification was done to determine which of the activity changes in chPeAFPV1 or chPeAFPV3 was prevalent.

Table 1 shows the predicted physicochemical properties of AFPs. chPeAFPV6 and chPeAFPV7 showed a slightly higher pI value (8.57 and 8.25, respectively) than PeAfpB (7.8) but none of them reached the pI value of PeAfpA (9.48). Regarding net charge at pH 7, chPeAFPV7 showed a similar charge (+ 2.5) to that of PeAfpB (+ 2.2) while chPeAFPV6 net charge was higher (+ 4.2), but it did not reach the significantly higher net charge of PeAfpA (+ 8.7).

**Table 1:** Physicochemical properties of antifungal proteins. Molecular mass (MM), Ɛ_280_, isoelectric point (pI) and grand average hydropathy (GRAVY) are theoretical measures obtained from ProtParam tool of the ExPASy Proteomics Server (Gasteiger et al. 2005). Net charge at pH 7 is a theoretical measure obtained from Protein Calculator v3.4 (http://protcalc.sourceforge.net)

Protein	aa	MM (kDa)	ε_280_	pI	Net charge (pH 7)	GRAVY	Reference
PeAfpA	57	6.64	0.673	9.48	8.7	−1.08	(Garrigues et al. 2018, Giner-Llorca et al. 2023a)
PeAfpB	58	6.69	0.668	7.80	2.2	−1.47	(Garrigues et al. 2018, Giner-Llorca et al. 2023a)
chPeAFPV1	58	6.67	0.670	8.27	2.9	−1.48	Giner-Llorca et al. 2023a
chPeAFPV3	58	6.66	0.671	7.77	1.7	−1.53	Giner-Llorca et al. 2023a
chPeAFPV6	58	6.69	0.668	8.57	4.2	−1.48	This work
chPeAFPV7	58	6.64	0.674	8.25	2.5	−1.54	This work

The newly designed chPeAFPV6 and chPeAFPV7 were produced in *P. chrysogenum* Δ*paf* strain as previously described (Giner-Llorca et al. 2023a), with yields of 34 mg/L and 24 mg/L for chPeAFPV6 (clone PCGL45431) and chPeAFPV7 (PCGL45554), respectively. Both proteins migrated similarly to PeAfpB in SDS-PAGE and reacted with the anti-PeAfpB antibody but not with the antibody raised against PeAfpA in western blot analyses. The identity of both AFPs was successfully verified by peptide mass fingerprinting (LC–ESI–MS/MS) (Fig. S1).

The antifungal activity of parental and newly designed AFPs was tested, side by side, against a wide selection of filamentous fungi and yeasts (Table 2, Fig. 1C and Fig. S2). For better comparison, antifungal activity of the previously designed chimeras chPeAFPV1 and chPeAFPV3 is also shown (Giner-Llorca et al. 2023a). PeAfpA inhibits the growth of all tested species with MICs ranging from 1 to 32 µg/mL (0.15 to 4.8 µM), while PeAfpB demonstrated moderate potency against some species and lack of activity against *Magnaporthe oryzae, Aspergillus westerdijkiae, Fusarium oxysporum* and all the yeasts evaluated (Table 2). Notably, we observed that PeAfpA activity was strain-dependent against *Candida glabrata* and *Candida auris* (Table 2). chPeAFPV6 showed an intermediate behaviour between both parental proteins with MICs ranging from 1 to > 64 µg/mL (0.15 to > 9.6 µM). chPeAFPV6 was as potent as PeAfpA against all the PeAfpB-sensitive species, clearly improving the PeAfpB activity, and it was inactive against the PeAfpB-tolerant species with the exception of *A. westerdijkiae* and *Cryptococcus neoformans* that were effectively inhibited by chPeAFPV6 (Table 2). By contrast, in the conditions tested, chPeAFPV7 was an inactive protein, even against *Byssochlamys spectabilis*, previously described as a highly AFP-sensitive fungus which was inhibited by the typically inactive chPeAFPV3 (Table 2) (Giner-Llorca et al. 2023a).

**Table 2:** Minimum inhibitory concentration (MIC) (µg/mL) of antifungal proteins. chPeAFPV1, chPeAFPV3, chPeAFPV6 and chPeAFPV7 are abbreviated as V1, V3, V6 and V7, respectively. Asterisks (*) indicate that MICs of V1 and V3 are extracted from previous studies (Giner-Llorca et al. 2023a); n.d.: not determined

Microorganism	PeAfpA	PeAfpB	V6	V7	V1*	V3*
*Penicillium digitatum* CECT 20796	1	8	1	> 64	1	> 64
*Penicillium expansum* CECT 20906	2	32	2	> 64	2	> 64
*Penicillium chrysogenum* Q176	1	8	1	> 64	n.d	n.d
*Penicillium roqueforti* CECT 2905	2	8	2	> 64	n.d	n.d
*Botrytis cinerea* CECT 20516	2	32	2	> 64	4	> 64
*Byssochlamys spectabilis* CECT 2983	1	4	1	> 64	1	16
*Magnaporthe oryzae* PR9	16	> 64	> 64	> 64	> 64	> 64
*Aspergillus westerdijkiae* CECT 2948	1	> 64	2	> 64	n.d	n.d
*Fusarium oxysporum* 4287	4	> 64	> 64	> 64	> 64	> 64
*Saccharomyces cerevisiae* BY4741	8	> 64	> 64	> 64	> 64	> 64
*Aspergillus fumigatus* ATCC 46645	4	8	4	> 64	n.d	n.d
*Cryptococcus neoformans* H99	2	> 64	8	> 64	n.d	n.d
*Candida albicans* SC5314	32	> 64	> 64	> 64	n.d	n.d
*Candida glabrata* 4012	4	> 64	> 64	> 64	n.d	n.d
*Candida glabrata* 1384	16	> 64	> 64	> 64	n.d	n.d
*Candida auris* B121040	4	> 64	> 64	> 64	n.d	n.d
*Candida auris* B12406	4	> 64	> 64	> 64	n.d	n.d
*Candida auris* B12663	8	> 64	> 64	> 64	n.d	n.d
*Candida auris* B17201	32	> 64	> 64	> 64	-	-

### Antifungal activity against *P. digitatum* does not correlate with cell permeabilisation or ROS induction

**Fig. 2:**
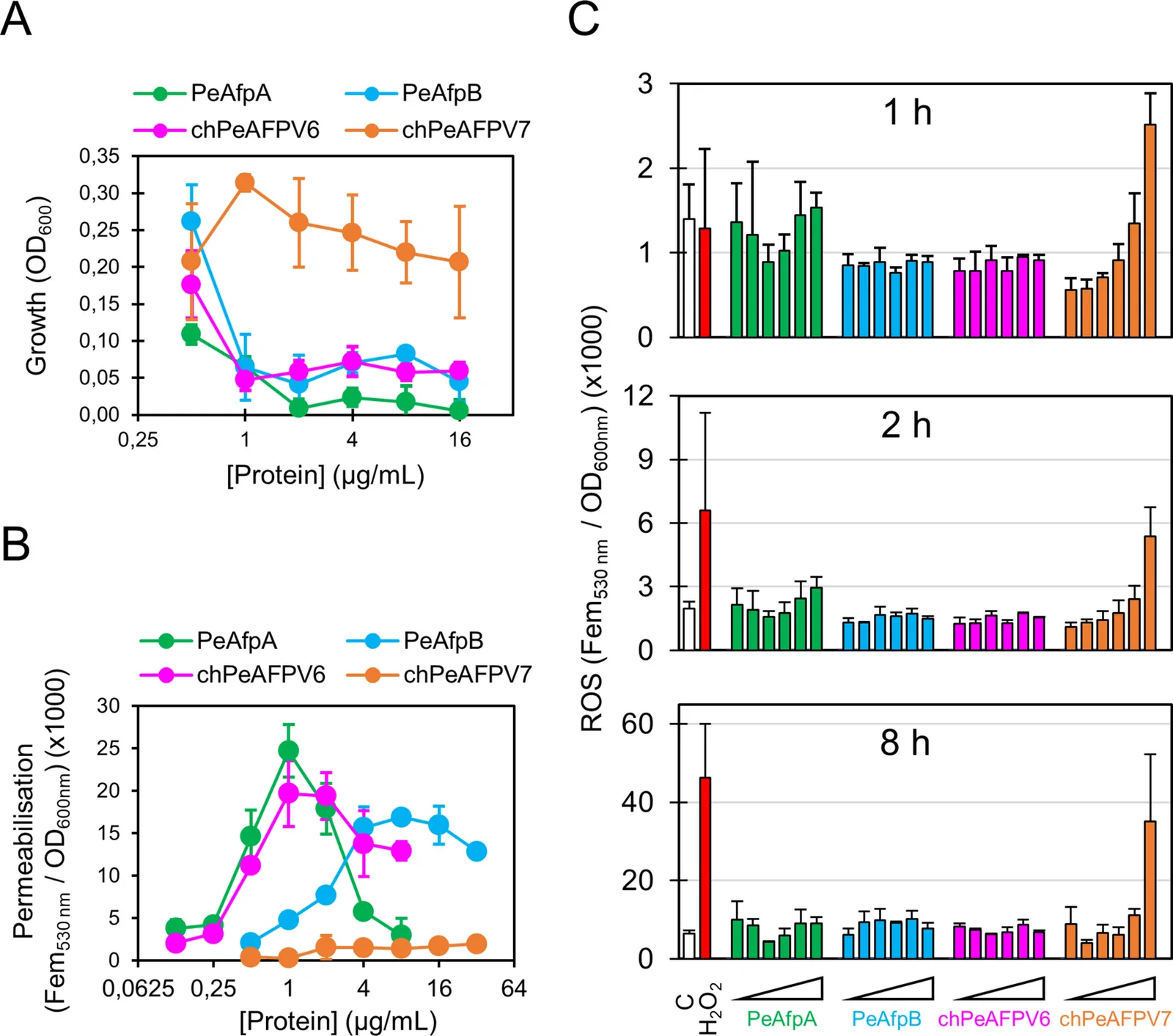
Comparative analysis of fungal growth, cell permeabilisation and ROS production upon treatment with PeAfpA, PeAfpB, chPeAFPV6 and chPeAFPV7. **A** Dose–response curves showing the inhibitory activity of each protein against *P. digitatum* germlings. **B** SYTOX Green uptake assay to evaluate cell permeabilisation upon treatment with each protein at different concentrations. **C** ROS determination with the fluorescent probe CM-H2DCFDA after treatment with different concentrations of each protein. Relative fluorescence (RFU) was measured after 8 h of treatment (**B**) and after 1 h, 2 h and 8 h of treatment (**C**) by first subtracting fluorescence values of the control condition (no protein) and then dividing by the growth (OD600) of each well. Data points and error bars represent means and SD, respectively, of three technical replicates. All assays were repeated three times

Permeabilisation of plasma membrane and ROS induction have been linked to the activity of many antimicrobial peptides (Theis et al. 2003; Toth et al. 2016; van der Weerden et al. 2010). For these experiments, we treated pre-germinated germlings with AFPs to assess the permeabilisation and oxidative stress provoked by proteins on growing hyphae. Figure 2A shows the dose–response curves of AFPs against *P. digitatum* after 48 h of addition of proteins to germlings. Under these conditions, chPeAFPV7 remained inactive while PeAfpA, PeAfpB, and chPeAFPV6 were remarkably similar in activity, in contrast to that observed against *P. digitatum* conidia (Fig. 1C). The active PeAfpA, PeAfpB, and chPeAFPV6 induced permeabilisation of germlings, assessed by the fluorescent dye SYTOX Green (SG), in a concentration-dependent manner (Fig. 2B). However, the shapes of the curves were different as the fluorescence signal reached its peak at lower protein concentrations for PeAfpA and chPeAFPV6 compared to PeAfpB (Fig. 2B), and then declined, and therefore did not correlate across the proteins or with the inhibition curve (Fig. 2A). By contrast, the inactive chPeAFPV7 was unable to cause cell permeabilisation.

To evaluate whether oxidative stress is relevant for the antifungal activity, we used a fluorometric assay to measure ROS production upon treatment of *P. digitatum* germlings with the proteins, and included controls of both untreated and H_2_O_2_-treated hyphae (Fig. 2C). PeAfpA, which does not induce a burst of ROS (Giner-Llorca et al. 2024), was also used as an internal control. Similarly to PeAfpA, chPeAFPV6 and PeAfpB did not significantly induce ROS production across the full protein range (0.5 to 16 µg/mL), showing fluorescent signals similar to the untreated control. The H_2_O_2_-treated hyphae (50 mM) showed complete inhibition of growth comparable to the highest protein concentrations of the active AFPs and variants (data not shown). This H_2_O_2_ treatment provoked a burst of ROS production that was detectable after 2 h. Unexpectedly, the inactive chPeAFPV7 induced the highest ROS production at the maximum concentration, comparable to the inhibited H_2_O_2_-treated samples (Fig. 2C). These findings suggest that ROS production is not involved in the mode of action of parental and newly designed AFPs.

### Crystal structure of chPeAFPV6 and chPeAFPV7

**Fig. 3:**
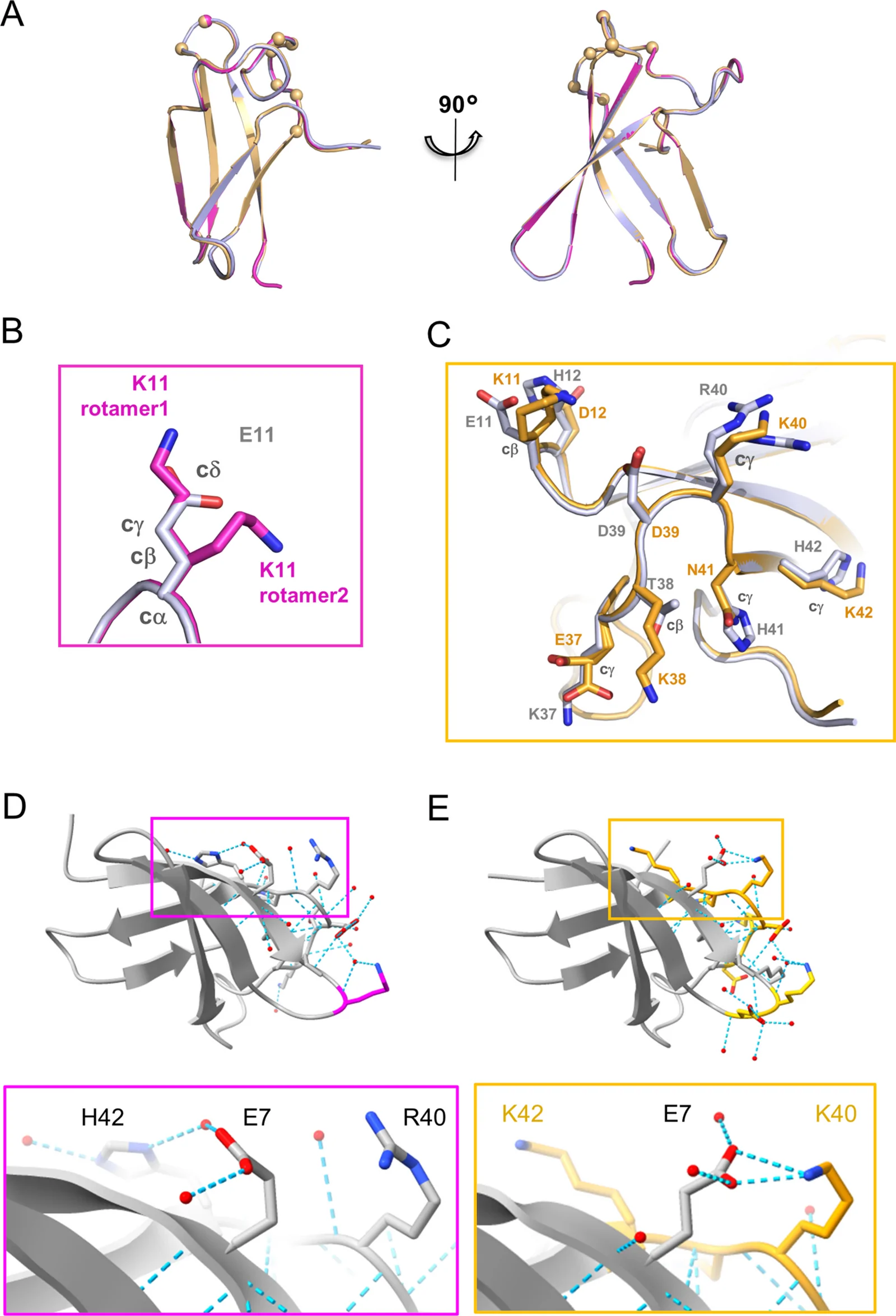
Structural characterisation of the newly designed chPeAFPV6 and chPeAFPV7. **A** Two orthogonal views of the overlapping structures of PeAfpB (PDB ID: 7ZTF) (Giner-Llorca et al. 2023a), chPeAFPV6 (9FQF) and chPeAFPV7 (9FQG) in cartoon representation with close views of chPeAFPV6 (**B**) and chPeAFPV7 (**C**) mutated residues. A close view of the alternative conformation of the E7 side chain in chPeAFPV6 (**D**) and chPeAFPV7 (**E**) is shown. Structures are coloured in blue for PeAfpB, magenta for chPeAFPV6 and orange for chPeAFPV7

In order to understand the molecular basis of the different antifungal activity exhibited by chPeAFPV6 and chPeAFPV7, we solved their crystal structures. In both cases, crystals belong to space group P3_2_21 and diffract to atomic resolution (1.2–1.3 Å) with one monomer in the asymmetric unit (Table S2). The two structures solved in this study were compared with those of PeAfpB, chPeAFPV1 and chPeAFPV3 previously solved (Giner-Llorca et al. 2023a). Their overall conformation is identical to PeAfpB structure, with five antiparallel β-strands arranged in two β-sheets that are disposed orthogonally building a β-barrel (Giner-Llorca et al. 2023a) (Fig. 3A).

Main chain superposition from PeAfpB and chPeAFPV6 gives a root mean square deviation (RMSD) of only 0.09 Å, reflecting the extremely high structural similarity between both molecules that differ in just one amino acid substitution (E11K) (Fig. 3A). A close view of the chPeAFPV6 residue K11 shows a double conformation (Fig. 3B). Comparison with the PeAfpB structure shows that in one of the conformations the atoms Cβ, Cγ and Cδ from K11/E11 side chains are superposed, and Cε from K11 is in the same position as Oε from E11. In the other conformation only the Cβ from K11/E11 are superposed (Fig. 3B).

In the case of chPeAFPV7, where substituted amino acids represent 13.5% of the protein, RMSD with PeAfpB is also extremely low with values of 0.29 Å for the whole molecule (Fig. 3A). In the substituted loops, the position of the main-chain is also almost identical with RMSD of 0.3 Å for L1 and 0.21 Å for L3. If we compare in detail each amino acid in the substituted loops, we find that not only the main-chain Cα atoms occupy the same positions, but also all Cβ and γ from residues 12, 40, 41 and 42 and even Cδ for residue 37 superpose perfectly although the side-chains are different (Fig. 3C).

These observations corroborate our previous findings that alterations in the loops of PeAfpB do not affect its overall conformation (Giner-Llorca et al. 2023a), thereby establishing this protein as an ideal scaffold for amino acid substitutions in order to confer specific antifungal properties. Detailed structural comparisons of the newly designed proteins further reveal that these modifications not only influence the substituted side-chains but also induce allosteric effects on distant, non-modified residues. The analyses of the structures of chPeAFPV6 (active) and chPeAFPV7 (inactive) revealed an alternative conformation of the side chain in the E7 residue, which was not changed in our protein designs (Fig. 3D-E). In the inactive protein chPeAFPV7, the carboxyl groups of E7 are making a strong salt bridge with the substituted K40 (Fig. 3E), while in the active chPeAFPV6 the side chain of E7 adopts an alternative conformation by rotating its carboxyl group 180 degrees, moving away from the native R40, and making a salt bridge with H42 (Fig. 3D). This alternative conformation of E7 also discriminates the active PeAfpB and chPeAFPV1 from the inactive chPeAFPV3 previously described (see PDB IDs: 7ZTF, 7ZUT and 7ZVH). This observation reveals a potential interaction between the γ-core motif and the L3 that might be important for antifungal activity.

### Internalisation of PeAFPs is neither sufficient nor essential for inhibiting *P. digitatum*

We analysed the binding and internalisation dynamics of PeAfpB, chPeAFPV6 and chPeAFPV7 in *P. digitatum* morphotypes. PeAfpA, which requires internalisation into mature hyphae of the fungus prior to exert its fungicidal activity (Giner-Llorca et al. 2024), was included as internal control. For this, we labelled these proteins with the fluorophore BODIPY™ FL EDA, which did not affect their antifungal activity (Fig. S3).

First, we analysed the interaction of the labelled proteins with conidia of *P. digitatum* (Fig. S4). There was no significant difference in the pattern of interaction of the proteins, showing a behaviour similar to the previous report with PeAfpA (Giner-Llorca et al. 2024), with discrete patches of protein accumulation in small quiescent conidia that changed to a more uniform distribution in swollen conidia, but no protein internalisation even after long incubation times (3 h). The only noticeable observation in conidia was the faint signal in the inactive chPeAFPV7.

**Fig. 4:**
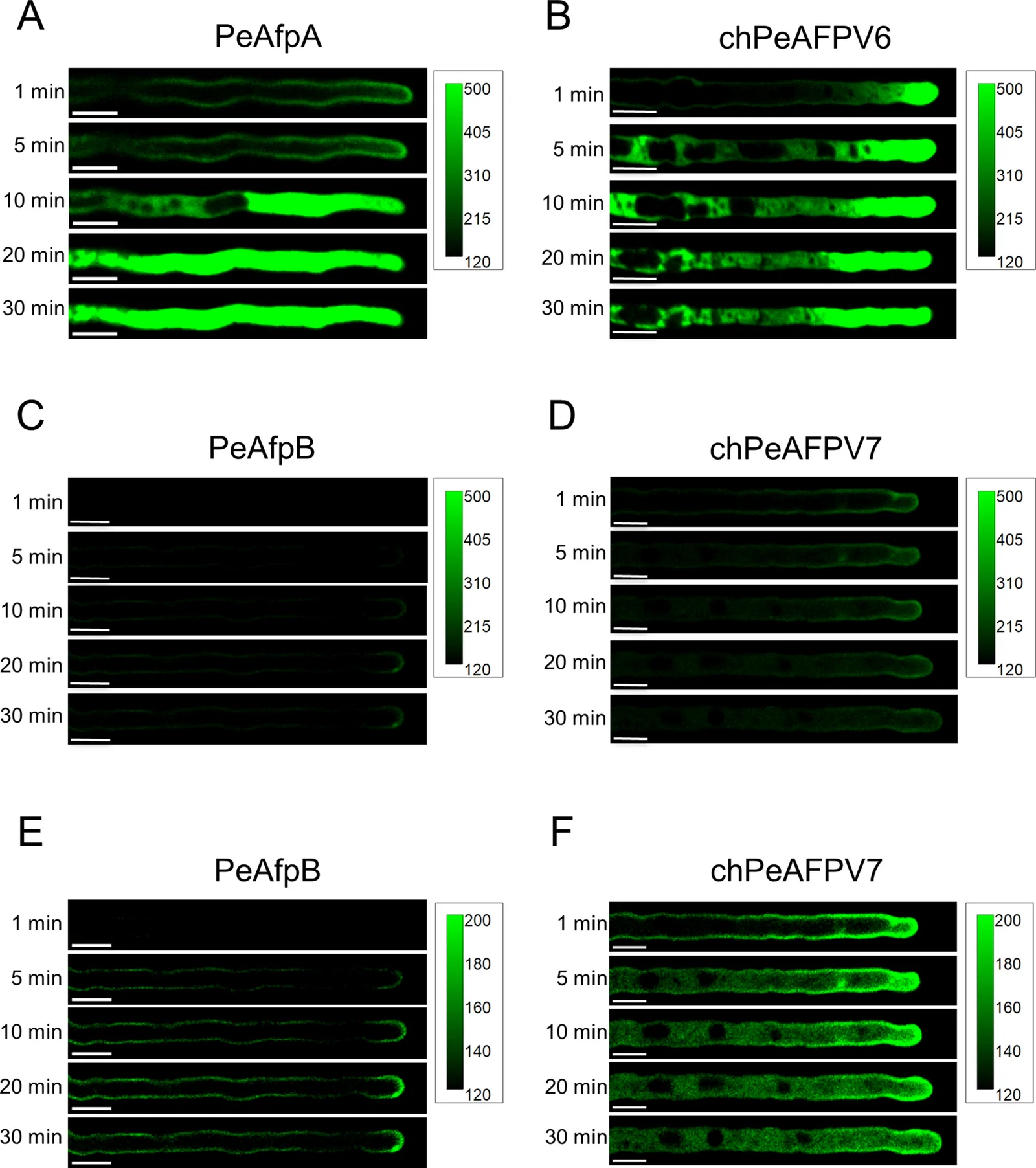
Internalisation dynamics of BODIPY-labelled AFPs in *P. digitatum* hyphae. Confocal laser microscopy images showing the time course of the interaction of BODIPY-labelled PeAfpA (**A**), chPeAFPV6 (**B**), PeAfpB (**C**) and chPeAFPV7 (**D**) (8 µg/mL) with *P. digitatum* germlings. **E** and **F** correspond to (**C** and **D**), respectively, with enhanced contrast to improve visualisation. Scale bars = 10 µm. Each calibration bar ranges from the minimum to the maximum intensity present in these images

Regarding the interaction with germlings, PeAfpA bound very rapidly to the outer surface of hyphae and a sudden internalisation at hyphal tips that led the protein to occupy the entire cytoplasm, excluding the vacuoles, was observed (Fig. 4A), in agreement with previous studies (Giner-Llorca et al. 2024). The same behaviour was noticed for the potent chPeAFPV6 (Fig. 4B), although its progress through the cytoplasm was slower than that of the parental PeAfpA (Fig. 4A-B). Both proteins inhibited hyphal growth at the concentration tested (Fig. 4A-B). The moderately active PeAfpB and the inactive chPeAFPV7 showed a substantially lower binding to *P. digitatum* hyphae that could only be clearly observed by adjusting the intensity range of the images (see the calibration bars in Fig. 4C-F). PeAfpB labelled the outer surface of hyphae, with higher signal being detected in the hyphal tip, but internalisation was not observed at the time frame studied, despite PeAfpB inhibiting hyphal growth at the concentration tested (Fig. 4C, E). Surprisingly, the dynamics of binding and internalisation of the inactive chPeAFPV7 was similar to PeAfpA and chPeAFPV6 albeit with much lower binding. However, chPeAFPV7 internalisation was not enough to inhibit hyphal growth (Fig. 4D, F).

### PeAfpA penetrates *C. auris* cells in a cell-wall dependent process

**Fig. 5:**
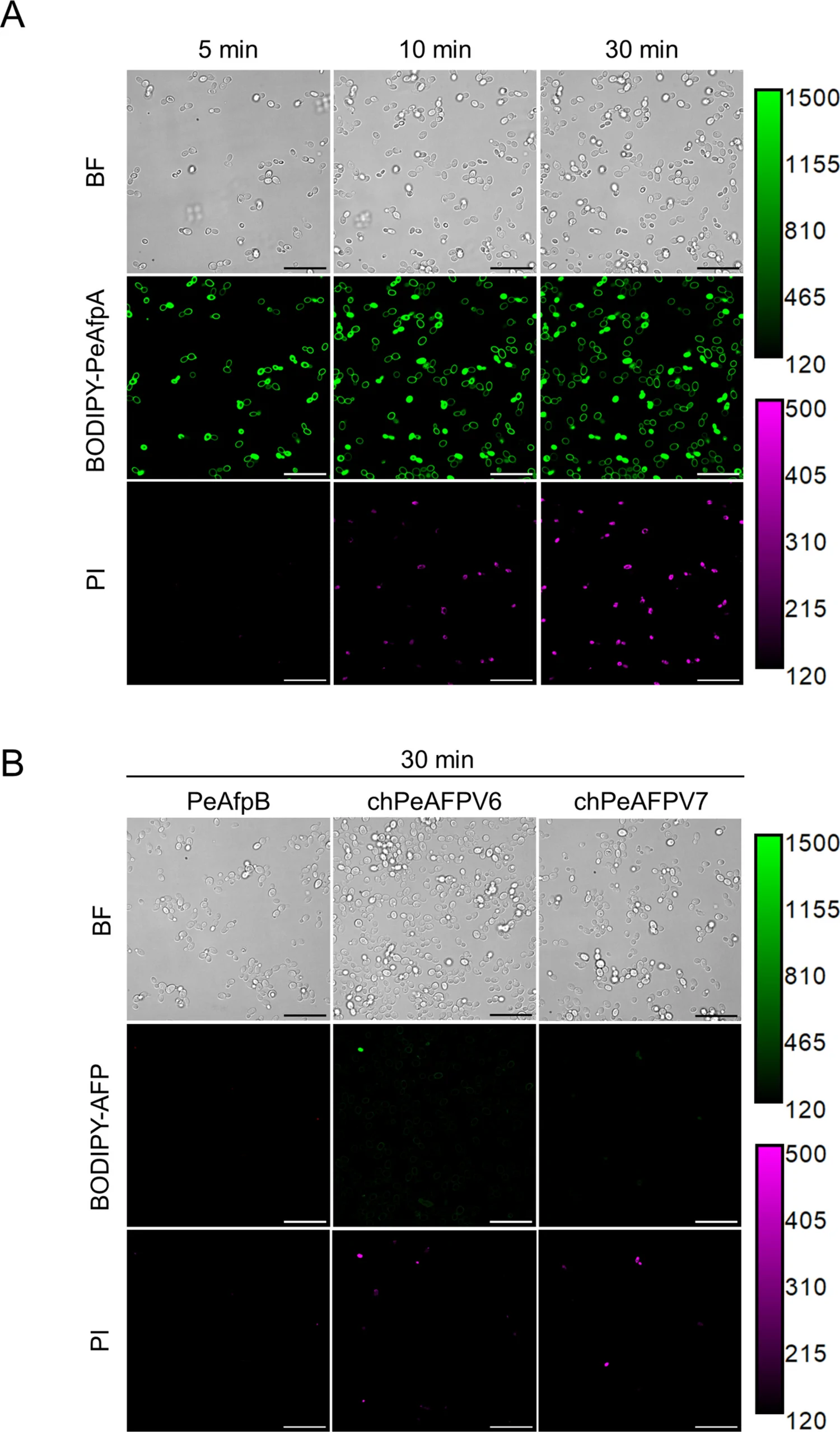
PeAfpA is able to internalise and kill *C. auris* cells. **A** Confocal laser scanning microscopy images showing the time course of the interaction and the internalisation of BODIPY-PeAfpA (8 µg/mL) in *C. auris* cells. **B** Images showing the lack of interaction between *C. auris* and the rest of the tested proteins (PeAfpB, chPeAFPV6 and chPeAFPV7). Propidium Iodide (PI) has been used as a cell death marker. Scale bar = 30 µm. Calibration bars range from the minimum to the maximum intensity present in each channel

With the aim of comparing the dynamics of interaction and internalisation of parental and newly designed proteins between two distantly related fungi, we extended the study to the yeast human pathogen *C. auris* (Fig. 5). In the first five minutes, BODIPY-PeAfpA homogeneously labelled the yeast cell surface and started to internalise in some of the cells. After 10 min and 30 min of exposure, more cells with the cytosol labelled were observed. At these time points, not all the cells intracellularly labelled with the protein showed Propidium Iodide (PI) staining, suggesting that protein internalisation occurs before cell death, as previously described for *P. digitatum* (Giner-Llorca et al. 2024). No binding of PeAfpB and chPeAFPV7 to yeast cells was observed after 30 min of incubation (Fig. 5B), while very faint fluorescence was detected for chPeAFPV6. Internalisation was not observed either, while PI staining was barely observed in a negligible percentage of cells, which we presume that were already dead before the treatment (Fig. 5B). These results are in accordance with the antifungal activity assays, which showed that these AFPs were inactive against *C. auris* (Table 2).

**Fig. 6:**
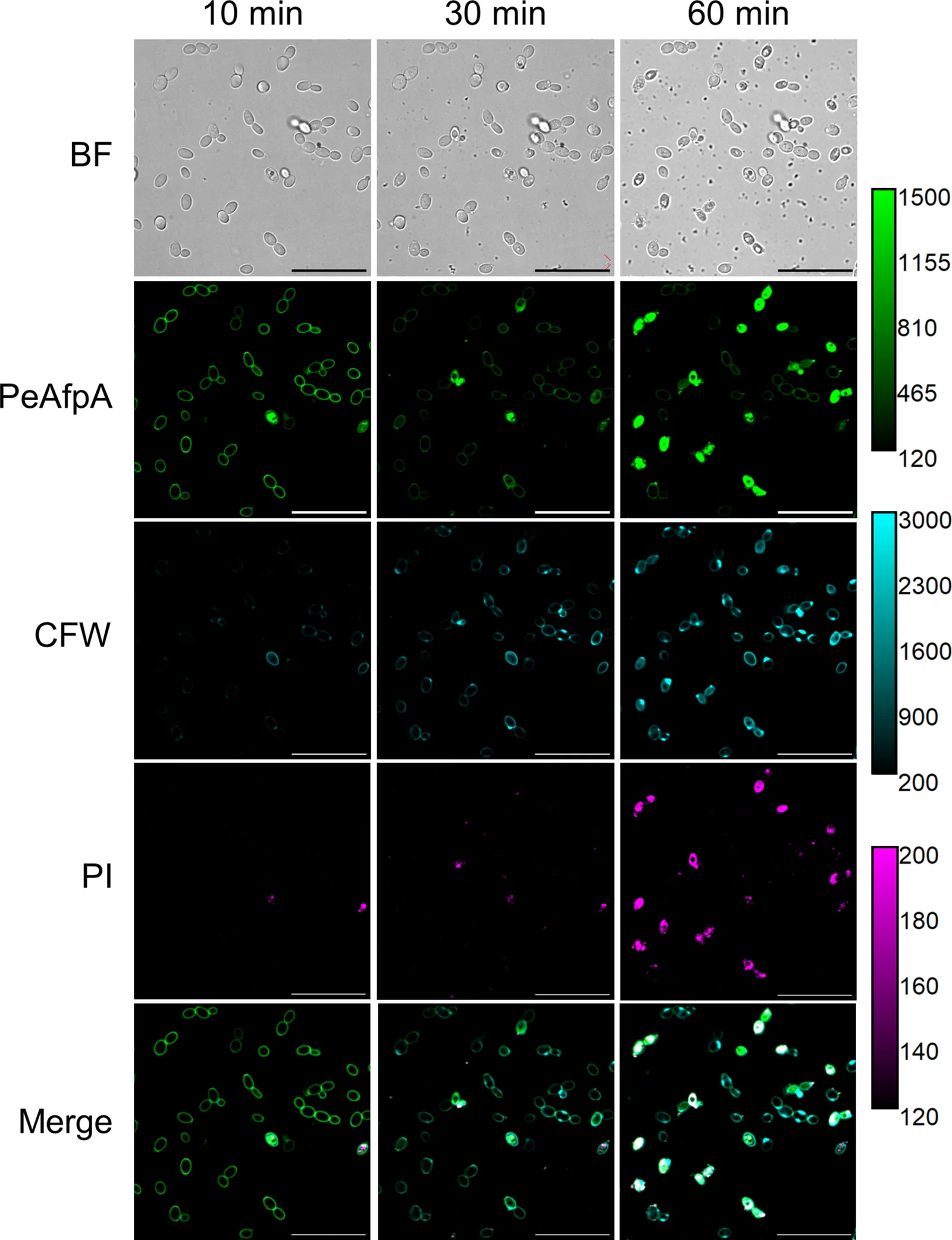
PeAfpA co-localises with CalcoFluor White (CFW) in *C. auris* cell wall prior to internalisation and cell death. Panels show confocal laser microscopy images of the BODIPY-PeAfpA (8 µg/mL) interaction with *C. auris* cells treated with 50 µg/mL CFW and 20 µg/mL propidium iodide (PI). Scale bar = 30 µm. Calibration bars range from the minimum to the maximum intensity present in these images

To gain insight into the relevance of CW in the mode of action of PeAfpA, we treated cells with CalcoFluor White (CFW) in combination with BODIPY-PeAfpA and PI (Fig. 6). Interestingly, we observed that CW staining with CFW became brighter in a time-dependent manner, while the PeAfpA signal at the cell envelope became weaker over time (Fig. 6), coupled with an apparent slowing in PeAfpA internalisation. If we compare with Fig. 5A, PeAfpA was able to penetrate several *C. auris* cells in 10 min in the absence of CFW, and PI staining was observed after 30 min, while *C. auris* cells showed a strong PI signal at 60 min in the presence of CFW (Fig. 6).

## Discussion

Based on the previous highly active chPeAFPV1 and the inactive chPeAFPV3 (Giner-Llorca et al. 2023a), in the present study we designed two new variants and characterised them, providing novel insights into the relevance of sequence motifs and the mode of action of AFPs, including the important and somewhat controversial aspects of protein internalisation and ROS production.

Changes in loops L1 and L3 of PeAfpB to obtain chPeAFPV6 and chPeAFPV7 were tolerated and both proteins were successfully produced, confirming both the suitability of the PeAfpB scaffold for introducing sequence modifications and the versatility of the *P. chrysogenum* expression system for producing AFPs and AFP sequence variants (Giner-Llorca et al. 2023a; Sonderegger et al. 2017, 2016, 2018). Besides, the 3D structure of both newly designed proteins revealed an almost identical conformation to PeAfpB, which is not limited to the β-strands but also extends to the loops. Therefore, the remarkable differences found in this study are directly related to the amino acid residues exchanged.

Previous studies demonstrated the importance of the conserved γ-core as a protein motif in which targeted amino acid substitutions influence antimicrobial activity of AFPs (Batta et al. 2009; Giner-Llorca et al. 2023a; Sonderegger et al. 2018). Single residue mutation of cationic residues in this domain abolished activity (Batta et al. 2009). However, in order to increase antifungal potency, the mutation of several residues that increased net charge was needed (Giner-Llorca et al. 2023a; Sonderegger et al. 2016). Here, the change of only one residue in the loop L1 within the γ-core of PeAfpB (E11K; Fig. 1) to render chPeAFPV6 enhanced the potency of the moderately active PeAfpB to that of PeAfpA and also had a profound effect to increase the internalisation capacity of chPeAFPV6 into *P. digitatum* hyphae. Therefore, our data demonstrate that the γ-core of PeAfpB is involved not only in the antifungal activity but also in the internalisation dynamic. Moreover, this improvement was markedly species-dependent. Although chPeAFPV6 and PeAfpB present a 98% sequence identity, chPeAFPV6 enhanced PeAfpB activity against the *Penicillium* and *Aspergillus* species tested, as well as *B. cinerea*, *B. spectabilis* and *C. neoformans* (Table 2). However, this enhanced activity did not extend to *M. oryzae*, *F. oxysporum* or yeast including *S. cerevisiae, C. albicans*, *C. glabrata* and *C. auris*. Accordingly, chPeAFPV6 was not able to interact and penetrate *C. auris* cells, contrarily to that observed with *P. digitatum* hyphae. These findings suggest that specific protein motifs are responsible for the distinct antifungal profiles of PeAFPs and that the anti-yeast activity of PeAfpA does not only rely on the γ-core motif.

chPeAFPV6 showed antifungal activity comparable to the previous chPeAFPV1, which contained two PeAfpA residues (K10 and D11) in the PeAfpB sequence (Giner-Llorca et al. 2023a), highlighting the relevance of the K residue alone. It is worth mentioning that this amino acid exchange rendered chPeAFPV6 with an increased net charge (4.2), notably higher than PeAfpB (2.2) and chPeAFPV1 (2.9) but still far from that of PeAfpA (8.7) (Table 1). It is assumed that the cationic nature of AFPs allows them to be electrostatically attracted to the negatively charged CW and plasma membrane (Marcos et al. 2008; Muñoz et al. 2013). However, our results indicate that positive net charge is not the unique determinant of antifungal activity, as previously described for chPeAFPV1 (Giner-Llorca et al. 2023a), NFAP2 (Varadi et al. 2023) and PAF (Sonderegger et al. 2018).

chPeAFPV7 combines the swap in loop L3 from the inactive chPeAFPV3 with the change in the L1 of the γ-core from the highly active chPeAFPV1 (K10 and D11) (Giner-Llorca et al. 2023a) (Fig. 1), and provides novel insights into the contributions of different protein motifs to full antifungal activity. Results show that substitutions in L1 do not reverse the loss of activity due to the swaps in loop L3 (Table 2), suggesting that the L3 motif overrides the effect of the γ-core. However, chPeAFPV7 was able to internalise into *P. digitatum* hyphae under the experimental conditions tested, confirming that the substitutions done within the γ-core have a significant impact on internalisation capacity. This finding points to the possibility of using variants with the substituted γ-core motif as internalisation shuttles.

Both L1 and L3 are in close proximity (Fig. 1B) and our structural data confirm that, although the mutations do not affect the protein scaffold, changes in one loop affect the exposed side chains in the other (Fig. 3). These changes are not restricted to the substituted residues but also include allosteric effects on neighbouring residues, particularly E7 within the γ-core. While further work is needed to confirm our observations, the structural information provided suggests that mutations in L3 promote hydrogen bonding with E7 that may affect the protein interaction surface beyond the specific substitution site, and consequently, its antifungal activity.

Our results suggest a correlation between antifungal activity and *P. digitatum* permeabilisation, since the active PeAfpA, PeAfpB and chPeAFPV6 caused a concentration-dependent increase in SG uptake by *P. digitatum* hyphae, while the inactive chPeAFPV7 was unable to permeabilise the fungal cell. However, the shapes of the permeabilisation curves determined by SG uptake and the growth inhibition curves of germlings did not correlate for the active AFPs and variants, making it unclear whether cell permeabilisation is the cause or consequence of cell death. Previously, we showed that the internalisation of PeAfpA precedes cell death and permeabilisation and, therefore, that the translocation of the protein across membranes does not cause per se permeabilisation (Giner-Llorca et al. 2024). This is also the case of the inactive chPeAFPV7, whose internalisation does not cause permeabilisation. Cell permeabilisation has been proposed as a primary mode of action for some AFPs as the distant *A. fischeri* NFAP2 (Toth et al. 2016), which would act primarily through its pore-forming ability to disrupt membrane integrity through selective lipid binding (Pavela et al. 2024). Permeabilisation may be also the final cell death marker of a more complex antifungal mechanism.

Oxidative stress as determined by ROS production has been linked to the primary mechanism of some AFPs (Bugeda et al. 2020; Holzknecht et al. 2020; Huber et al. 2020; Leiter et al. 2005; Virágh et al. 2015). In this context, our study has also provided relevant and novel clues. Previously, we showed that the killing mechanism against fungal hyphae of PeAfpA did not correlate with ROS production (Giner-Llorca et al. 2024). Here, we did not detect internalisation of the parental PeAfpB although it successfully inhibited hyphal growth, permeabilised fungal cells and did not induce ROS production. Regarding new variants, we found that chPeAFPV6, which resembles the antifungal activity and permeabilisation capacity of PeAfpA, also behaved like PeAfpA regarding internalisation pattern and absence of ROS production. Finally, chPeAFPV7 was able to internalise into *P. digitatum* hyphae, although it has no antifungal activity and does not permeabilise membranes, but substantially induces ROS production in the conditions tested. The fact that chPeAFPV7 can penetrate the cell without causing permeation or inhibiting growth suggests that it does not alter by itself the plasma membrane. Regarding ROS production, our results suggest that it does not seem to be related with the killing mechanism. It could be hypothesised that, in the case of chPeAFPV7, the fungus might be able to overcome the intracellular effect of the protein by a ROS-dependent defence mechanism. Overall, results show a positive correlation between antifungal activity and cell permeabilisation that does not apply to protein internalisation or ROS production. Our results shed a new light on the future research in the mode of action of AFPs and open the possibility of using these sequence variants to identify the determinants of binding, internalisation and antifungal activity, as well as the intracellular targets involved in cell killing.

This work expands the antifungal profile of PeAfpA previously described (Garrigues et al. 2018; Giner-Llorca et al. 2023a; Martínez-Culebras et al. 2021). Here we show that the protein effectively kills a selection of human fungal pathogens (Table 2), which are currently considered as priority pathogens by the World Health Organisation (WHO) (WHO 2022). Regarding the mechanism of action against the critical human pathogen *C. auris*, PeAfpA is a cell-penetrating protein that needs to internalise in order to exert its inhibitory activity, correlating with our previous observations with *S. cerevisiae* (Giner-Llorca et al. 2023b) and *P. digitatum* (Giner-Llorca et al. 2024). Similarly, it has been shown that PAF, PAFB (Huber et al. 2020) and PAFC (Holzknecht et al. 2020) need to be taken up into *C. albicans* cells to induce cell death. By contrast, PeAfpB and the newly designed proteins were not able to effectively bind or penetrate *C. auris* cells and were inactive under the conditions tested. This is in marked contrast with the results observed with *P. digitatum* and therefore reinforces the existence of different binding and/or internalisation mechanisms among filamentous fungi and yeast that affect the specificity of AFPs and need to be further studied.

We have also demonstrated that the CW is involved in the interaction of PeAfpA with *C. auris* since CFW addition seems to interfere with this process, similarly to that observed with *P. digitatum* hyphae (Giner-Llorca et al. 2024). Our previous transcriptomic study on *S. cerevisiae* also points out the importance of CW and CW-related signalling in the mechanism of PeAfpA (Giner-Llorca et al. 2023b). The CW relevance for the mode of action of AFPs has also been evidenced by the characterisation of *P. digitatum* strains lacking genes involved in mannosylation of CW proteins or chitin synthesis (Bugeda et al. 2020; Gandia et al. 2019; Giner-Llorca et al. 2023a; Harries et al. 2015).

In summary, this study proves that the PeAfpB scaffold stands as a promising tool for protein engineering and custom design (or design *a la carte*) where different functional motifs modulating antifungal activity, membrane permeabilisation and ROS production can be combined without perturbing its structural stability or its suitability for recombinant production. This work provides novel insights about the mode of action of AFPs through characterisation of rationally designed variants. We show how a single amino acid exchange in the γ-core can drastically and specifically enhance the antifungal potency against filamentous fungi but not against yeasts and enable internalisation into *P. digitatum* hyphae, offering new tools to identify species-specific determinants in AFPs and design new variants as internalisation shuttles. It is shown that the surface-exposed loop L3 of the PeAfpB scaffold may play a significant role for determining antifungal activity through interaction with the ɣ-core. The ROS production observed with the inactive chPeAFPV7 and the low levels in the active PeAfpA, PeAfpB and chPeAFPV6 suggest that oxidative stress is either specific to certain AFPs or just a fungal stress defence mechanism not related with killing. This study shows that internalisation is neither sufficient nor essential for causing *P. digitatum* cell death. Overall, results evidence that the mode of action of parental PeAFPs and variants exhibit similarities but also important differences, pointing the modularity of these proteins and different mechanisms of action (possibly strain-specific), and offer new tools to test hypotheses to solve in detail the killing mechanism of these promising antifungal molecules.

## Supplementary Information

Below is the link to the electronic supplementary material.

ESM 1 Supplementary Material 1 (PDF 1.80 MB)

## Data Availability

All data generated or analysed during this study are included in this published article and the public repository DIGITAL CSIC (10.20350/digitalCSIC/18103).
