# Supplementary material for: Engineering Penicillium expansum antifungal proteins unveils new clues about their mode of action
Source: Appl Microbiol Biotechnol. 2026 Apr 20;110:154. doi: 10.1007/s00253-026-13782-5 (PMC13197446; doi:10.1007/s00253-026-13782-5)
Supplement: Supplementary file 1 [file 253_2026_13782_MOESM1_ESM.pdf]

Supplementary material Giner-Llorca *et al*.

Engineering *Penicillium expansum* antifungal proteins unveils new clues about their mode of action

Moisés Giner-Llorca[a], Francisca Gallego del Sol[b], Stefani de Ovalle[a], Darren D. Thomson[c], Elaine M. Bignell[c], Alberto Marina[b], Jose F. Marcos[a], Paloma Manzanares[a],*

a Department of Food Biotechnology, Instituto de Agroquímica y Tecnología de Alimentos (IATA), Consejo Superior de Investigaciones Científicas (CSIC), Paterna, Spain.

b Instituto de Biomedicina de Valencia (IBV), CSIC and CIBER de Enfermedades Raras (CIBERER), Valencia, Spain

c MRC Centre for Medical Mycology, University of Exeter, Exeter, UK.

*Corresponding author

E-mail: pmanz@iata.csic.es (Paloma Manzanares)

Supplementary Figure S1 Giner-Llorca et al.

A

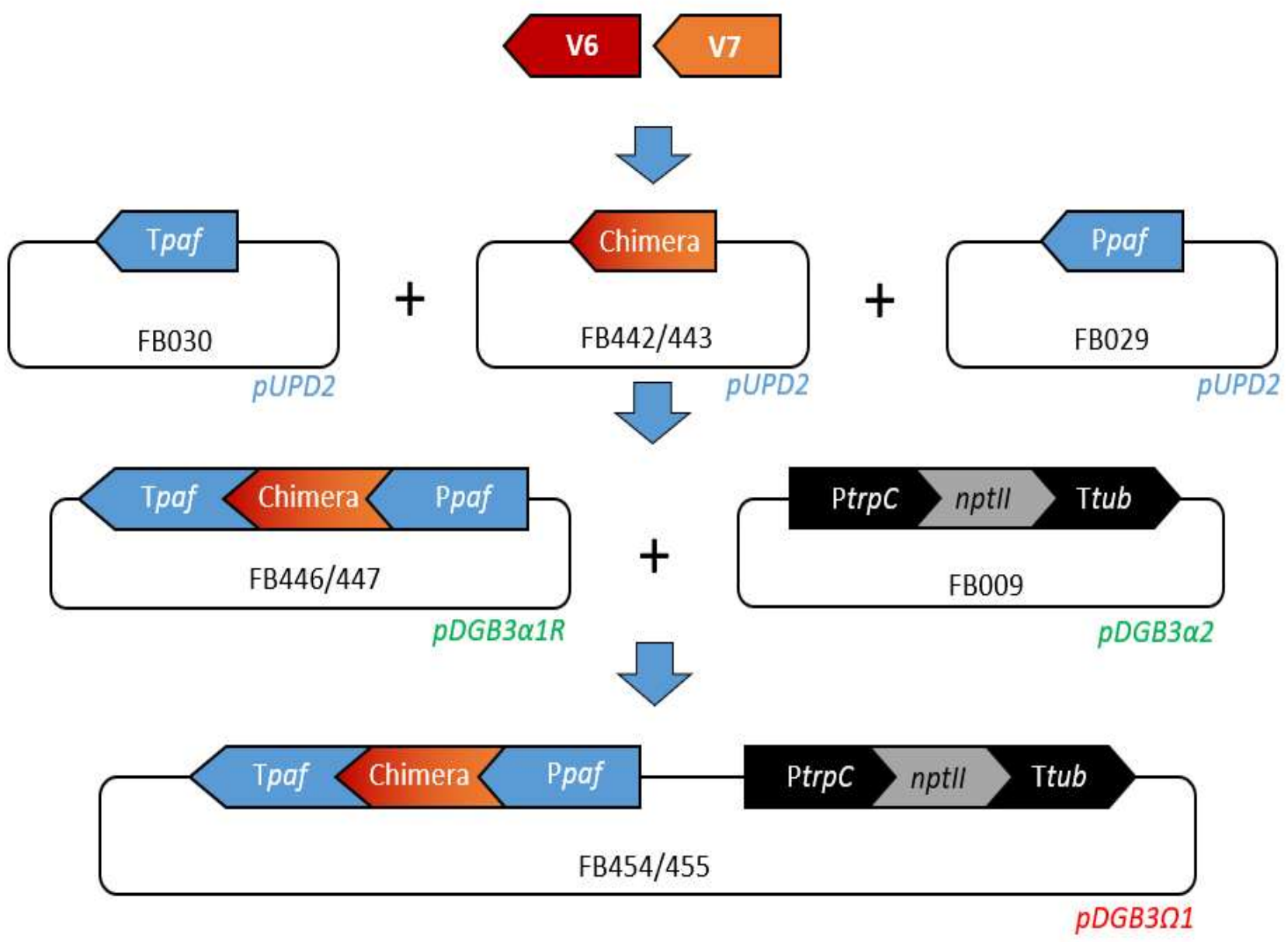

B

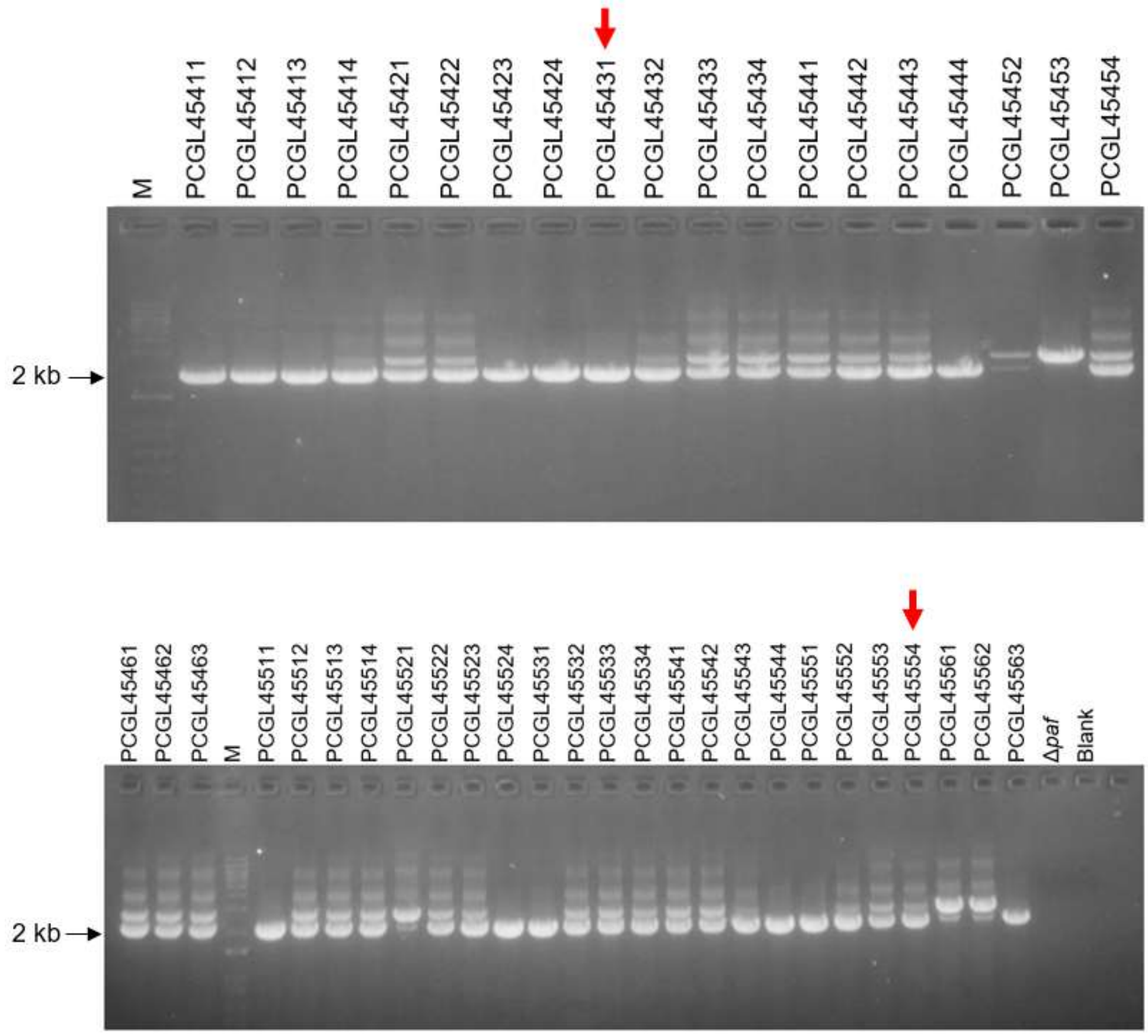

C

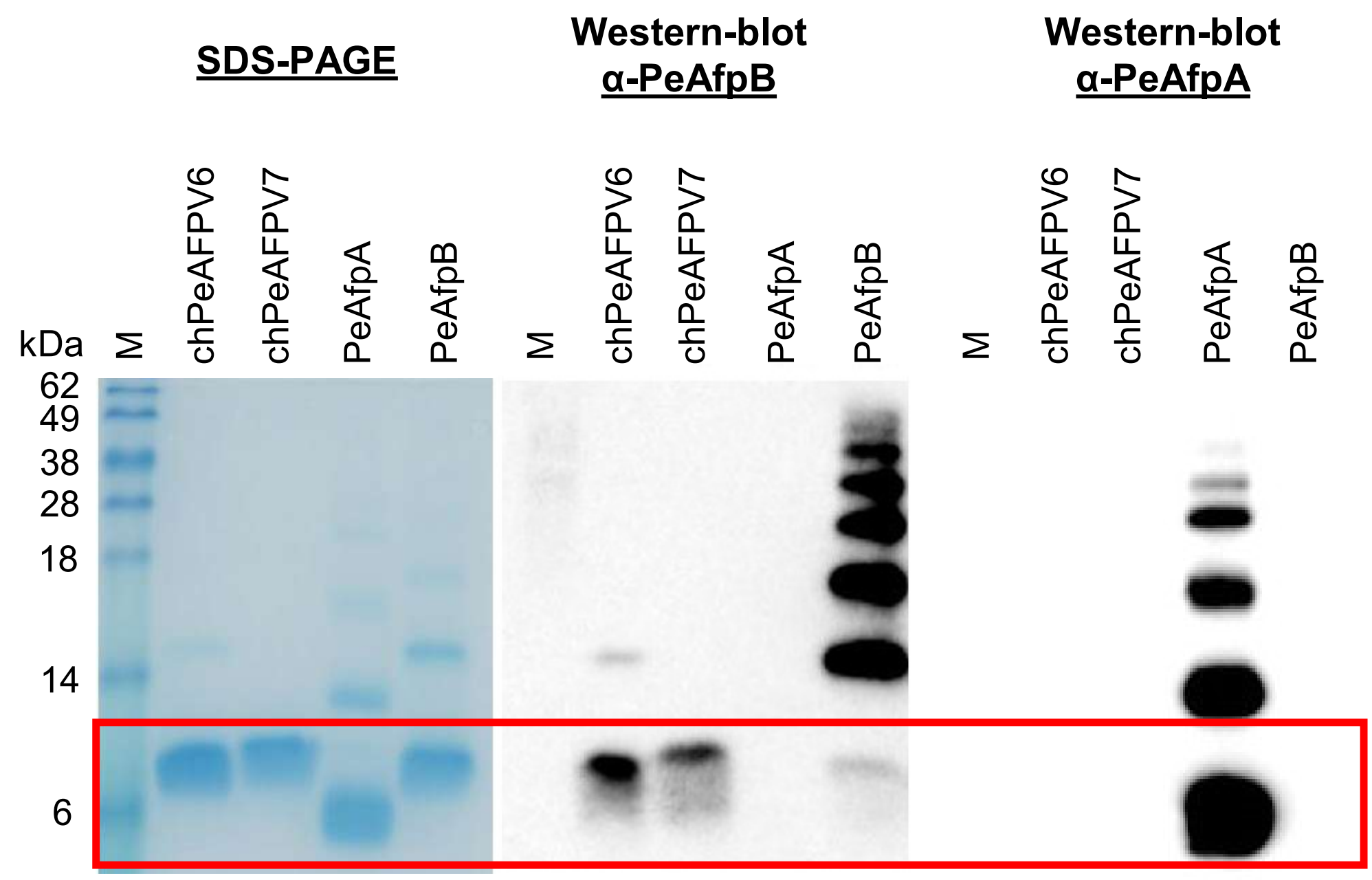

D

```
PeAfpA:     -VLYTGQCFKKDNICKYKVNGKQNIAKCPSAANKRCEKDKNKCTFDSYDRKVTCDFRK

PeAfpB:     LSKYGGECSKEHNTCTYRKDGKDHIVKCPSADNKKCKTDRHHCEYDDHHKTVDCQTPV

chPeAFPV6:  LSKYGGECSKKHNTCTYRKDGKDHIVKCPSADNKKCKTDRHHCEYDDHHKTVDCQTPV
(69%)       LSKYGGECSK
            LSKYGGECSKK
            LSKYGGECSKKHNTCTYR
               YGGECSKKHNTCTYR
                      KHNTCTYR
                              KDGKDHIVK
                              KDGKDHIVKCPSADNK
                               DGKDHIVKCPSADNKKCK
                                  DHIVKCPSADNKKCKTDR
                                       CPSADNKKCKTDR
                                              KCKTDR

chPeAFPV7:  LSKYGGECSKKDNTCTYRKDGKDHIVKCPSADNKKCEKDKNKCEYDDHHKTVDCQTPV
(100%)      LSKYGGECSKK
            LSKYGGECSKKDNTCTYR
               YGGECSKK
               YGGECSKKDNTCTYR
                      KDNTCTYR
                              KDGKDHIVKCPSADNKK
                               DGKDHIVKCPSADNKKCEK
                                  DHIVKCPSADNKKCEK
                                       CPSADNKKCEK
                                       CPSADNKKCEKDK
                                       CPSADNKKCEKDKNK
                                              KCEKDKNK
                                                    NKCEYDDHHKTVDCQTPV
                                                      CEYDDHHKTVDCQTPV
                                                              TVDCQTPV
```

Fig. **S1** Production, purification and identification of chimeric PeAFPs. (A) Schematic diagram of the modular assembly of each chimera genetic sequence into pUPD2 vectors with FB030 and FB029 (paf promoter and terminator sequences, respectively) to obtain pDGBα1R plasmids FB446 and FB447 and a subsequent binary assembly of these plasmids with FB009 to obtain final pDGBΩ1 vectors FB454 and FB455 for *P. chrysogenum* transformation. (B) PCR confirmation of *P. chrysogenum* clones transformed for the recombinant production of chimeras chPeAFPV6 and chPeAFPV7. Primers used were OJM483 (P*paf*) and OJM484 (T*paf*) (Table S2). *P. chrysogenum* parental strain Δ*paf* was used as negative control. Red arrows indicate strains selected for protein production. (C) SDS-PAGE gel of the purified chPeAFPs (2 µg per sample) and their immunodetection with α-PeAfpA and α-PeAfpB. Two µg of pure PeAfpA and PeAfpB were also added as controls. A red rectangle is used to indicate the position of the proteins. (D) Peptide mass fingerprinting (PMF) of chimeras. In brackets, percentage (%) of primary sequence covered by PMF. Amino acids exchanged are highlighted in red (chPeAFPV6) and orange (chPeAFPV7).

Supplementary Figure S2 Giner-Llorca et al.

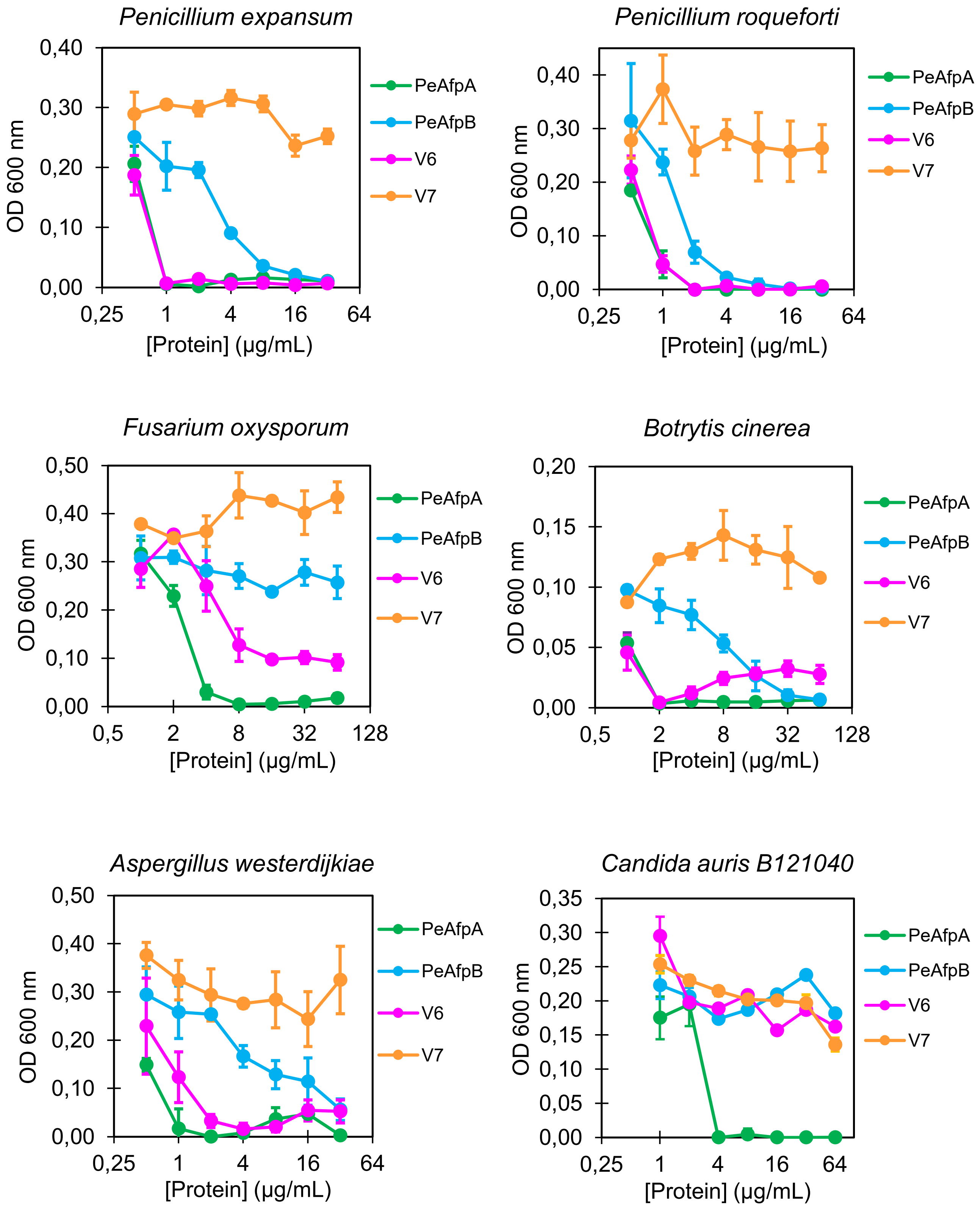

Fig. S2 Antifungal activity of natural and chimeric PeAFPs. Selection of dose-response curves showing the in vitro inhibitory activity of PeAfpA, PeAfpB, chPeAPV6 and chPeAFPV7 against filamentous fungi and yeasts. Curves show mean ± S.D. OD600 of triplicate samples after 72 h at 25ºC for filamentous fungi and 48 h at 37ºC for *Candida auris*.

Supplementary Figure S3 Giner-Llorca et al.

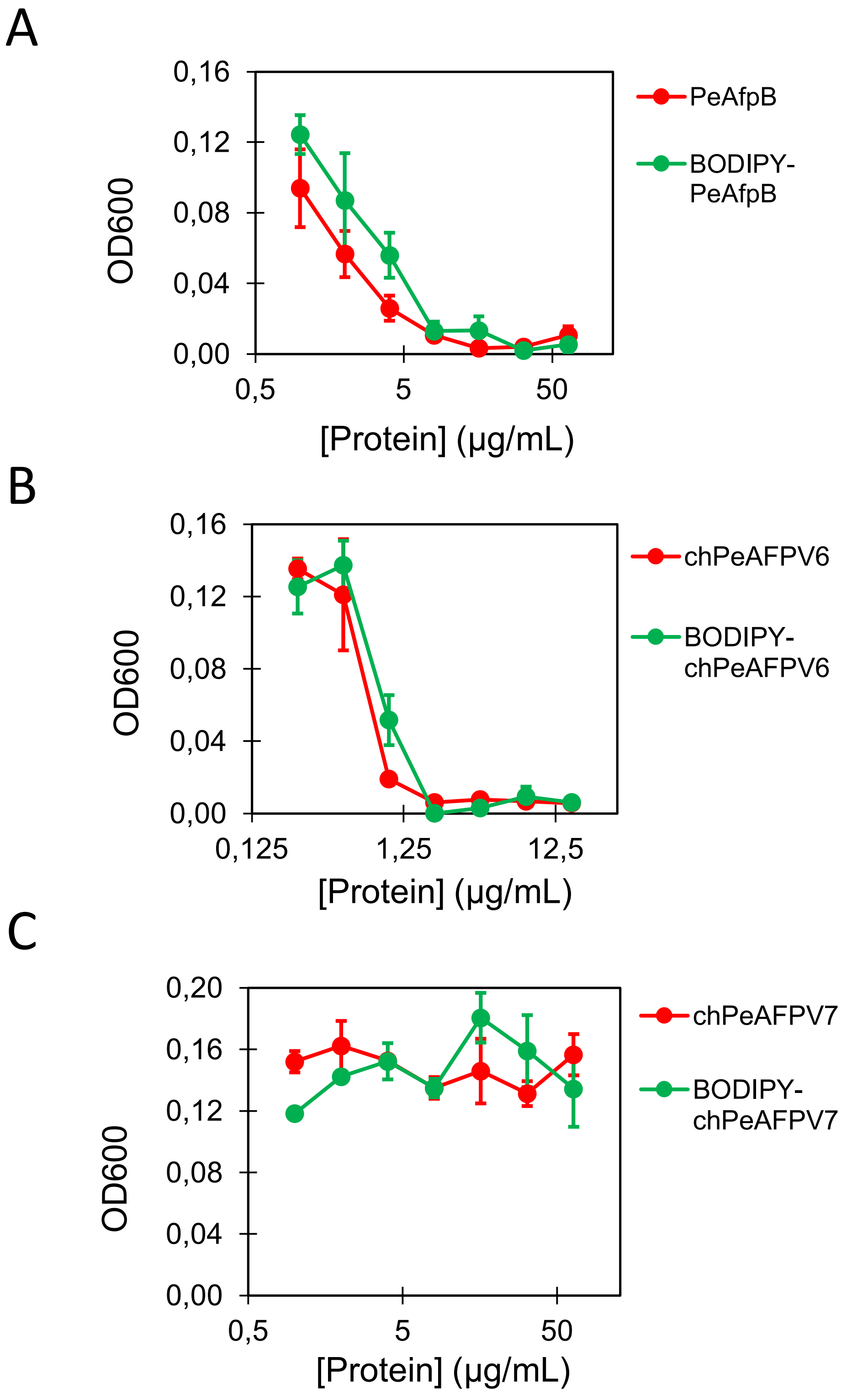

Fig. **S3** Comparison of *in vitro* antifungal activity between unlabelled and BODIPY-labelled AFPs. Panels show dose-response curves comparing the antifungal potency of PeAfpB with BODIPY-PeAfpB (A), chPeAFPV6 with BODIPY-chPeAFPV6 (B) and chPeAFPV7 with BODIPY-chPeAFPV7 (C). Curves show mean and SD of triplicate values of the OD_{600} measurement at 72 h. All assays were repeated at least twice.

Supplementary Figure S4 Giner-Llorca et al.

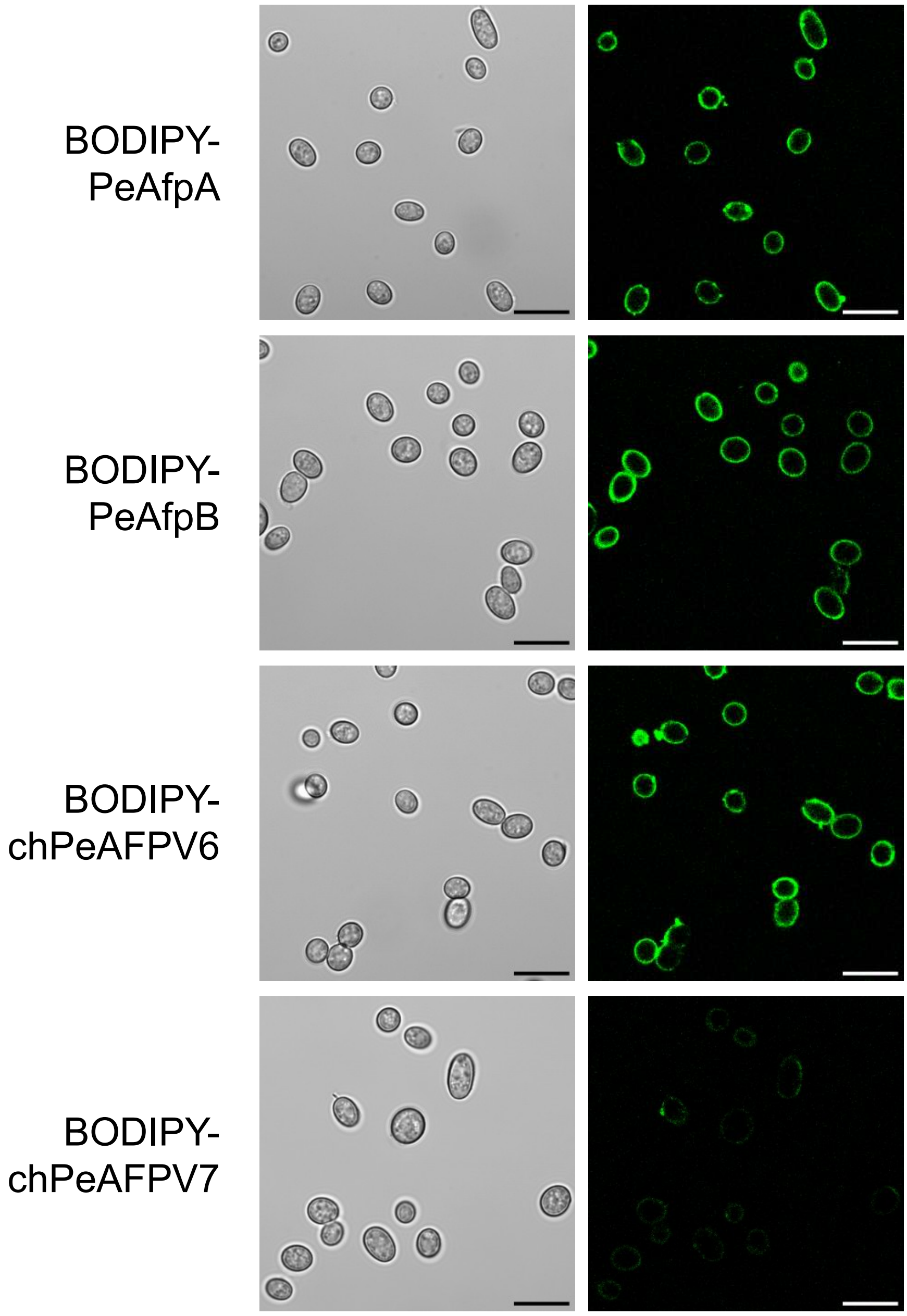

Fig. **S4** Comparison of the interaction of natural and chimeric AFPs with *P. digitatum* conidia. Confocal laser scanning microscopy images showing the binding of BODIPY-labelled proteins (8 µg/mL) to *P. digitatum* conidia after 1,5 h of treatment. Scale bar = 10 µm.

Table S1. FungalBraid (FB) elements used and FB vectors generated in this study.

Code	Genetic element	GB plasmid	Reference
FB009	P*trpC*::*nptII*::T*tub*	pDGB3α2	Hernanz-Koers *et al*., 2018
FB029	P*paf*	pUPD2	Hernanz-Koers *et al*., 2018
FB030	T*paf*	pUPD2	Hernanz-Koers *et al*., 2018
FB442	*chPeAFPV6*	pUPD2	This work
FB443	*chPeAFPV7*	pUPD2	This work
FB446	P*paf*::*chPeAFPV6*::T*paf*	pDGB3α1R	This work
FB447	P*paf*::*chPeAFPV7*::T*paf*	pDGB3α1R	This work
FB454	P*paf*::*chPeAFPV6*::T*paf*(←)::P*trpC*::*nptII*::T*tub*(→)	pDGB3Ω1	This work
FB455	P*paf*::*chPeAFPV7*::T*paf*(←)::P*trpC*::*nptII*::T*tub*(→)	pDGB3Ω1	This work

Abbreviations: Glyceraldehyde-3-phosphate dehydrogenase gene (*gpdA*); neomycin phosphotransferase gene (*nptII*) conferring geneticin resistance; tryptophan biosynthesis protein C gene (*trpC*); *β-tubulin* gene (*tub*).

Hernanz-Koers M, Gandía M, Garrigues S, Manzanares P, Yenush L, Orzaez D, Marcos JF. 2018. FungalBraid: A GoldenBraid-based modular cloning platform for the assembly and exchange of DNA elements tailored to fungal synthetic biology. Fungal Genetics and Biology 116:51-61. doi:10.1016/j.fgb.2018.04.010.

Table S2. Primers used for the molecular characterisation of *P. chrysogenum* transformants.

Name	Use	Sequence 5´- 3´	Tm (°C)	Origin	Reference
OJM197	F	CGTTAACTGATATTGAAGGAGCAT	66	P*trpc*	Garrigues *et al*., 2017
OJM483	F	ATCCCGGGGAATTCAGAGAGCTTTTCGTACG	62	P*paf*	Hernanz-Koers *et al*., 2018
OJM484	R	ATTCTAGAGCAGCAGTTTGATAGTTATCCCT	60	T*paf*	Hernanz-Koers *et al*., 2018
OJM524	F	GCTTTCGCTAAGGATGATTTCTGG	60	pUPD2	Hernanz-Koers *et al*., 2018
OJM525	R	CAGGGTGGTGACACCTTGCC	60	pUPD2	Hernanz-Koers *et al*., 2018
OJM533	F	CGAGTGGTGATTTTGTGCCG	60	pDGB3α/Ω	Hernanz-Koers *et al*., 2018
OJM534	R	CCCGCCAATATATCCTGTCAG	60	pDGB3α/Ω	Hernanz-Koers *et al*., 2018
OJM555	R	TCATCATGCAACATGCATGTA	58	T*tub*	Hernanz-Koers *et al*., 2018

Garrigues S, Gandía M, Popa C, Borics A, Marx F, Coca M, Marcos JF, Manzanares P. 2017. Efficient production and characterization of the novel and highly active antifungal protein AfpB from *Penicillium digitatum*. Scientific Reports 7:14663. doi:10.1038/s41598-017-15277-w.

Hernanz-Koers M, Gandía M, Garrigues S, Manzanares P, Yenush L, Orzaez D, Marcos JF. 2018. FungalBraid: A GoldenBraid-based modular cloning platform for the assembly and exchange of DNA elements tailored to fungal synthetic biology. Fungal Genetics and Biology 116:51-61. doi:10.1016/j.fgb.2018.04.010.

Table S3. Data collection and refinement statistics.

	**chPeAFPV6**	**chPeAFPV7**
**Data collection**		
Space group	$P3_221$	$P3_221$
Cell dimensions (Å)	a=b=45.96, c=41.89 α =β=90, γ=120	a=b=46.16, c=41.94 α =β=90, γ=120
Resolution (Å)*	41.9-1.2 (1.22-1.2) [a]	41.9-1.3 (1-32-1.3)
Unique reflections	16405 (794)	12340 (603)
Completeness (%)	100 (99.8)	99.8(95.8)
Multiplicity	18.6 (14.7)	18.5(14.0)
I/σ(I)	21.3 (5.1)	30.3(4.0)
Rpim	0.02 (0.169)	0.011(0.183)
**Refinement**		
R_{work}	0.122 (0131)	0.138 (0,177)
R_{free}	0.145 (0.138)	0.167(0.210)
Mean B factors (Å^2)	15.0	22.0
Rmsd, bond (Å)	0.011	0.014
Rmsd, angles (°)	1.873	2.351
Monomers in ASU	1	1
**Ramachandran plot**		
Most favored (%)	98	98
Additional allowed (%)	2	2

* Values in parentheses are for highest-resolution shell.
